# Chinese Pirates

**Published:** 1882-05

**Authors:** 


### CHINESE PIRATES.

The towns contain a due number of tame cheats, but the bold, hectoring highwayman, the truculent sea robber, must be sought elsewhere. All along the Blue and Yellow rivers are found retail buccaneers, who hawk at a trifling quarry and fatten on slender profits. These poor rogues do not aspire to a ship of their own, they come paddling out of muddy creeks in the smallest of sampans, ill-armed, ill-clad, but plentifully smeared with fish-oil. If manfully confronted they fly; if grappled by the crews of the fourth-class junks, which they select as prizes, they slip like so many eels through the hands that grasp them, and their swimming makes amends for their lax courage.

Seldom do any very sinister results follow one of these attacks. If the fresh-water pirates prove victorious they are mild conquerors, and only too eager to be on shore again with their booty of rice and corn, stray garments, odd fragments of chain, bits of copper and brass hastily ripped from the poop and cabins, and, perhaps, the glorious trophy of a few rattling strings of cash.

The dollars and silver bars are generally too well hidden to be detected by such hurried searchers; food, rather than fortune, is the object of the foray; and, except in cases of remarkable temptation, no life is attempted and no torture resorted to.

With these amphibious, petty-larceny rogues the magistrates deal mildly, according to the traditions of Chinese justice. Three hundred strokes of the bamboo may be endured by the human frame. Four sleepless weeks in the "cangue," or bamboo pillory, may fail to madden a stolid, unimaginative coolie. A few minor tortures need only be added to these two first-named afflictions, and the culprit is thought to be most tenderly dealt with.

Pilferers in a fair or on the streets of a town are considered as still more venial offenders. A vigorous bastinado and a week of the pillory is the law's award in such trivial cases. Petty assaults are as leniently disposed of ; but fire-raising is a sin of the deepest die ; and the malicious piercing of a neighbor's dyke, to let in a devastating flood, is punished with extreme rigor. Murder and treasonable practices, wholesale piracy and armed brigandage, all cry aloud for death, more or less slow and painful, and parricide provokes the sternest chastisement of the Chinese, as it once did of the Roman law.—*All the Year Round.*

---

THE indignities offered the body of Pope Pius IX. lead the *Independent* to speak of the Papacy as of a light that is rapidly growing dim:

" We believe the present Pope to be one of the best Christians living, a man of uncommon good sense and intelligence. He is credibly reported to have hoped that he might soon break out from his quasi imprisonment and trust himself to the people and government of Rome. It is now believed that he will feel himself still a prisoner. It is hardly gracious, under these circumstances, to suggest that if the Italian government is indifferent and the Roman populace hostile, the Papacy has itself to thank for it; but it is true. Not Protestantism, or atheism, but the Roman Catholic Church has had the exclusive education of Italy and the control of Rome. This continued until the people were, by the policy of the Pope, utterly and finally alienated. Romanism, whether its religion or its policy, was faithfully tried and found wanting. All the failure, the whole of it, belongs to Romanism. If the people hate it, it is because it has taught them to do so. It taught the young, it repressed the old, it shut out 'error,' it did its own sweet will, with nobody to interfere, and the fruit is of its own sowing and raising. Not that we believe that Leo would have chosen to sow any such seeds as Pius and his predecessors did ; but the system did, and he must reap the fruit. Nor is it in good taste for Catholics to complain very loudly of the attitude toward their Pope of the inhabitants of the Holy City. Further, the vindictiveness of the Roman people toward the late Pope has a valid ground. He had been a temporal ruler, and such a one, too, as was not loved. His government was a tyranny. His prisons were filled with men who would have been free and eligible to honor in any but a tyrannously governed State. Some of these political prisoners still live, and hate the Papacy. The sons of others live and hate it. Their injuries do not justify their retaliation on his dead body; but they have good reason not to love the name of Pope. Still further, we do not see what there is so sacred about the person of a priest or a bishop or a pope that he should avoid the risks of performing his public duties because he is afraid of insult or death. The Vicar of Christ has no higher right of temporal authority in Rome than Christ had in Jerusalem. At the risk of his life, with a government that did not care to protect him, with a vicious element in the populace that was ready to stone or crucify him, he went daily to the Temple—the St. Peter's of Jerusalem—to teach. At the risk of his life he did this, and in doing it sacrificed his life. Why should a Pope be denied the same privilege of martyrdom?"

### CLAY-EATERS.

Much has been written about this practice, and various speculations and suggestions offered about "fatty clays" and "earths rich in organic matter," but recent research and experiment have shown that the mere presence of solid materials in the stomach is sufficient to allay the sensation of hunger for a time, so that in all probability these savages swallow the earth only to appease the cravings of nature until food can be obtained. The hunters and trappers of the far West make pills of calcined oyster shell and white of egg, which they swallow occasionally to stave off hunger and its disagreeable concomitants when on a long journey and their rations are exhausted. Tea or coffee would answer the purpose better, as, though affording no nourishment in themselves, they prevent the waste of tissue.

Electric lamps are extremely attractive to insects of various orders. The writer noticed clouds of them about the lights used for the illumination of Prospect Park and Niagara Falls last summer. Their shadows on the lawns and walks, sharply defined, gigantic, and crazily active, made a pantomime as fantastic and weird as was amusing. This artificial sunlight is likely to become a favorite hunting ground for the entomologist.

PROF. SCHLAGER, director of a noted insane asylum at Vienna, announces the result of experiments made by him in relation to the blue glass healing theory, which at one time attracted so much attention in America as well as abroad. He had a room furnished with windows of blue glass, and had the walls painted of the same color. He then selected sixty persons who were more or less deranged mentally, and made them the subjects of experimentation for a period of three years, placing them at selected times in the blue room and carefully noting the apparent effects upon them. He discovered that the abnormally aroused and excited temperament experienced a remarkably soothing and quieting influence in the blue light, and he expresses the conviction that with persons thus mentally deranged, with whom every other method of treatment has failed, this should be tried. He does not report any complete cures made by this means alone, but says that in most cases the treatment has proved beneficial, and that if continued systematically and persistently, the indications are that it will lead to complete restoration. In no case did it work injury. He expresses the intention to continue his experiments, and calls upon all associates and colleagues in the treatment of the insane to do the same, and make careful notes of their observations. Prof. Schlager has also made valuable and interesting experiments in treating deranged persons of abnormally depressed or sluggish and apathetic temperaments by exposing them in a similar manner to red light. His conclusions seem to be based upon careful and scrupulous study and observation, and are attracting deserved attention.

